# Transcriptomic data sets for *Salmonella enterica* serovar Typhimurium 14028S that survived ingestion by *Acanthamoeba castellanii*, hydrogen peroxide treatment, or starvation

**DOI:** 10.1128/mra.01093-24

**Published:** 2025-09-08

**Authors:** Alexander Balkin, Andrey Plotnikov, Tatiana Konnova, Elena Shagimardanova, Yuri Gogolev, Natalya Gogoleva

**Affiliations:** 1 Kazan Institute of Biochemistry and Biophysics, Kazan Scientific Center of Russian Academy of Sciences 242112 , Kazan, Russia; 2 Ural Branch of the Russian Academy of Sciences, Institute for Cellular and Intracellular Symbiosis 104696 , Orenburg, Russia; 3 Skolkovo Institute of Science and Technology 366033, Moscow, Russia; 4 Loginov Moscow Clinical Scientific Center 575342, Moscow, Russia; 5 Institute of Fundamental Medicine and Biology, Kazan Federal University 64922, Kazan, Russia; 6 Research Department for Limnology, Universität Innsbruck 73182, Mondsee, Austria; The University of Arizona, Tucson, Arizona, USA

**Keywords:** *Salmonella enterica*, *Acanthamoeba castellanii*, transcriptome, hydrogen peroxide treatment, starvation

## Abstract

*Salmonella* is a pathogenic bacterium that can survive in hostile environments and inside heterotrophic protozoan cells. Here, we present transcriptomic data for *Salmonella enterica* grown in a rich medium, cultured under starvation conditions, treated with hydrogen peroxide, and extracted from *Acanthamoeba castellanii* cells after 8 and 15 h of infection.

## ANNOUNCEMENT

The ability of *Salmonella* to spread via protozoa is one of the challenges in preventing salmonellosis outbreaks (1, 2). This paper presents combined RNA-seq data for *Salmonella* surviving in *Acanthamoeba* at 8 and 15 h post-infection (hpi), free-living bacteria in poor medium, rich medium, and upon hydrogen peroxide treatment, providing insight into their general and specific stress tolerance mechanisms.

*Salmonella enterica* serovar Typhimurium 14028S was obtained from ATCC in 2010 and stored at −70°C. Single colonies were obtained on lysogeny broth (LB) agar and transferred to LB tubes corresponding to at least four control and experimental replicates. The cultures were grown at 37°C with shaking (190 rpm) to mid-log phase (OD_600_ = 0.4) and either fixed directly (control), treated with 1 mM hydrogen peroxide for 20 min, or washed three times and cultured for 2 h in PAS medium (3) without shaking to induce starvation. For fixation, all the cultures were diluted with an equal volume of ice-cold RNA-stabilizing solution (19% ethanol, 1% acidic phenol, pH 5.5) and incubated on ice for 30 min, after which bacterial cells were harvested by centrifugation (4).

*Acanthamoeba castellanii* Neff (ATCC 30010) was purchased from ATCC and maintained in batch culture. Fresh *Acanthamoeba* cultures were grown for 7 days in PYG medium (5) at 27°C to cell density 4–5 × 10^5^ × mL^−1^. Cells were collected by centrifugation at 800×*g* for 5 min at 25°C, washed twice, and suspended in 10 mL of PAS. *Salmonella* cultures obtained from individual colonies were grown in LB for 8 h to stationary phase, after which the bacteria were harvested by centrifugation, washed twice with PAS, and added to the *Acanthamoeba* cultures at a bacteria:amoeba cell ratio of 100:1. After 1 h, the medium was supplemented with gentamicin to 100 µg × mL^−1^, and after an additional hour, it was replaced with PAS containing 10 µg × mL^−1^ gentamicin. At 8 and 15 hpi, the amoeba cells were collected by centrifugation, suspended in 10 mL of ice-cold RNA-stabilizing solution, supplemented with 0.2% SDS, and incubated for 30 min. After amoeba cell lysis, *Salmonella* cells were collected by centrifugation at 3,000×*g* and 4°C for 5 min.

Total RNA was extracted from the *Salmonella* pellets using RNA Extract Reagent (Evrogen, Moscow, Russia) and purified using the RNase-Free DNase I kit (Ambion, Austin, TX). The NEBNext rRNA Depletion Kit (Bacteria) (New England Biolabs, Ipswich, MA) was used to deplete rRNA from bacterial samples, and the Ribo-Zero Plus rRNA Removal Kit (Illumina, San Diego, CA) was used for bacterial-amoebic samples. Libraries were prepared using the NEBNext Ultra II Directional RNA Library Prep Kit for Illumina (NEB), assessed for correct sizing (300–350 bp) using a Bioanalyzer 2100 (Agilent, Santa Clara, CA), and quantified by qPCR using KAPA qPCR Library Quantification Kit (Roche, Basel, Switzerland). Single-end sequencing (100 bp) was performed on the NovaSeq 6000 instrument (Illumina).

**Fig 1:**
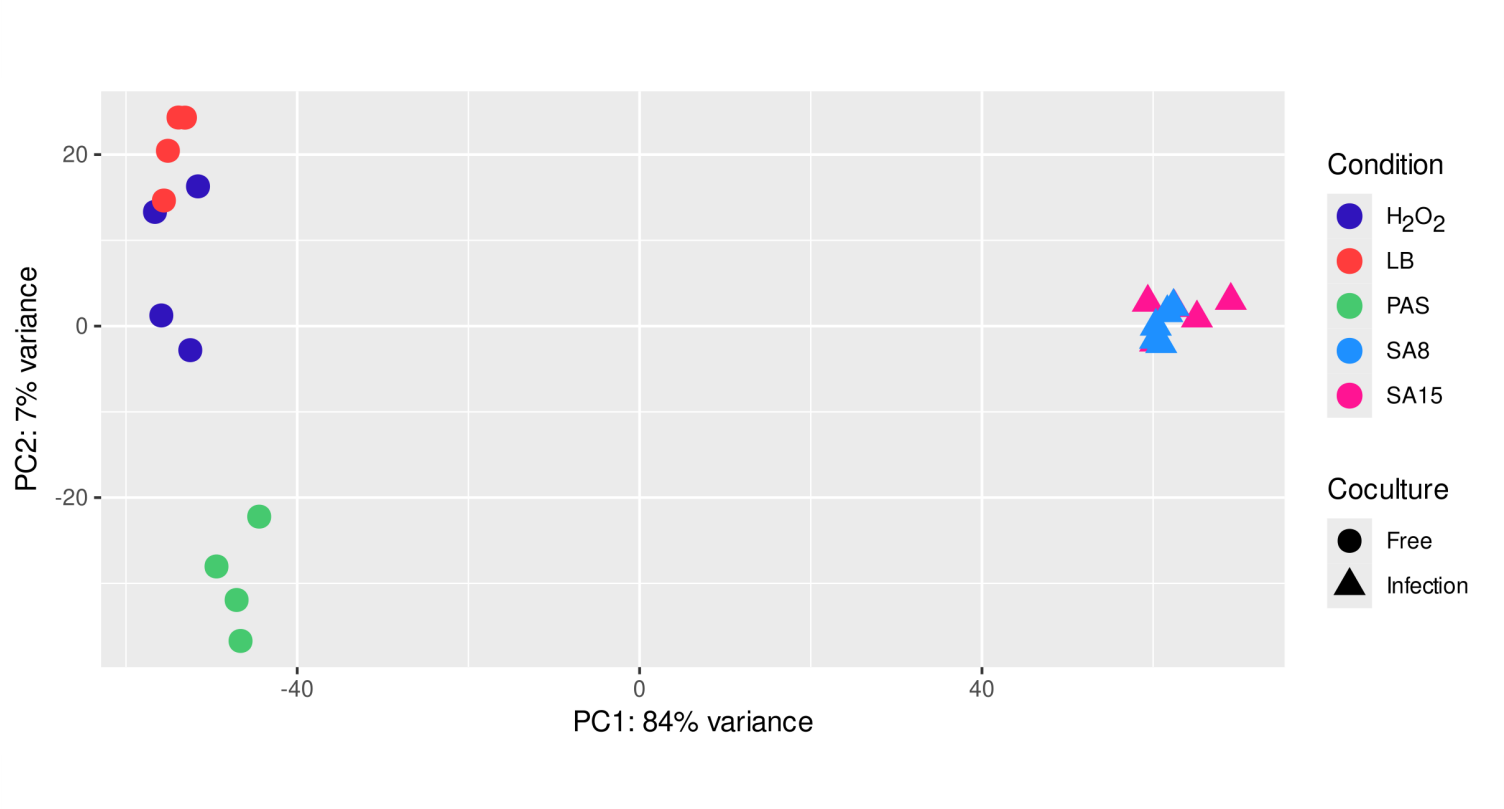
PCA plot of *Salmonella* Typhimurium 14028S RNA-seq data, showing distances between samples of *Acanthamoeba-Salmonella* infection (SA8 and SA15 correspond to 8 and 15 hours after infection), and free-living *Salmonella* cultures in a reach medium (LB), starved in a mineral medium (PAS), and treated with hydrogen peroxide (H_2_O_2_).

Reads were aligned to the *A. castellanii* genome (GCA_000313135) and then to the *S*. Typhimurium 14,028 s genome (GCA_000022165.1) (6) using STAR aligner v2.7.11a (7) (https://github.com/alexdobin/STAR) with the following options “--outFilterMultimapNmax 1,” “--sjdbGTFfeatureExon gene,”’, “‘--sjdbGTFtagExonParentTranscript gene_id,”’, “‘--sjdbGTFtagExonParentGene old_locus_tag,”’, “‘ --quantMode GeneCounts.”’. The featureCounts v1.4.6-p5 function of the Rsubread package version 2.14.2 (8) (http://subread.sourceforge.net) was used to count the number of reads mapped to each gene. A summary of reads and mapping results is present in Table 1. Principal component analysis (Fig. 1) was performed using DESeq2 (9).

**TABLE 1:** Summary of sequencing reads

Sample	No. of reads	No. of reads after rRNA removal	% of reads mapped to *Acanthamoeba* genome	% of reads mapped to *Salmonella* genome	GEO accession no.
LB1	11,677,968	9,951,088	0.03	97.75	GSM8346264
LB2	12,227,486	10,402,075	0.04	99.06	GSM8346265
LB3	12,186,198	10,339,993	0.03	99.24	GSM8346266
LB4	12,012,088	10,110,626	0.03	98.33	GSM8346267
PAS1	7,313,761	4,282,551	0.07	98.82	GSM8346268
PAS2	11,162,865	7,635,036	0.07	97.25	GSM8346269
PAS3	9,669,708	7,120,926	0.06	97.42	GSM8346270
PAS4	13,727,860	10,081,028	0.05	96.95	GSM8346271
H_2_O_2_1	12,292,846	7,182,528	0.06	97.20	GSM8346260
H_2_O_2_2	12,751,541	9,409,601	0.03	97.40	GSM8346261
H_2_O_2_3	12,204,911	9,346,880	0.04	96.75	GSM8346262
H_2_O_2_4	11,157,499	8,039,907	0.04	96.56	GSM8346263
SA8-1	194,425,176	29,814,977	31.96	39.54	GSM8346272
SA8-2	153,942,943	24,537,718	32.69	38.43	GSM8346273
SA8-3	161,012,096	26,322,001	31.44	40.08	GSM8346274
SA8-4	118,633,635	22,131,417	25.27	53.02	GSM8346275
SA8-5	157,915,032	21,658,283	34.55	34.17	GSM8346276
SA15-1	246,354,030	98,198,700	8.60	81.67	GSM8346277
SA15-2	56,135,189	18,662,502	9.28	81.58	GSM8346278
SA15-3	133,139,258	18,601,108	28.81	41.28	GSM8346279
SA15-4	141,461,121	23,041,425	21.25	56.46	GSM8346280
SA15-5	114,374,110	17,842,861	27.93	44.24	GSM8346281

## Data Availability

The data have been deposited in the NCBI Gene Expression Omnibus (GEO) and are accessible through GEO series accession no. GSE270532. This data set may be useful for researchers who study the molecular mechanisms of host-pathogen interactions, as well as those looking for ways to prevent foodborne infections.
